# Clinical Impact of Personalized Physician’s Education and Remote Feedback Via a Digital Platform on Glycemic Control: Pilot Randomized Controlled Trial

**DOI:** 10.2196/67151

**Published:** 2025-05-01

**Authors:** Jin Yu, Joonyub Lee, Yeoree Yang, Eun Young Lee, Seung-Hwan Lee, Jae-Hyoung Cho

**Affiliations:** 1 Division of Endocrinology and Metabolism, Department of Internal Medicine Seoul St. Mary’s Hospital, College of Medicine The Catholic University of Korea Seoul Republic of Korea; 2 Catholic Smart Health Care Center The Catholic University of Korea Seoul Republic of Korea

**Keywords:** type 2 diabetes mellitus, digital health, education, distance, remote patient monitoring, distance counseling

## Abstract

**Background:**

The digital education platform Doctorvice (iKooB Inc.) offers face-to-face physician-patient education during outpatient clinic visits, remote glucose monitoring, and the delivery of educational messages, and is expected to be effective for personalized diabetes care.

**Objective:**

This study aims to evaluate the effectiveness of the digital education platform for diabetes care by comparing cases that included both face-to-face education and remote monitoring with those that included only face-to-face education.

**Methods:**

This was a randomized clinical study conducted at the Diabetes Center of Seoul St. Mary’s Hospital. Participants were aged ≥19 years and had glycated hemoglobin (HbA_1c_) levels between 7.5% and 9.5%. In the intervention group, physicians used the digital education platform to provide face-to-face education at enrollment and at the 3- and 6-month visits, along with remote monitoring during the first 3 months of the 6-month study period. The control group received conventional outpatient education. Both groups completed questionnaires—assessing satisfaction with diabetes treatment, diabetes-related stress, and adherence to diabetes medication—at the beginning and end of the study. The primary endpoint was the change in HbA_1c_ levels.

**Results:**

A total of 66 participants were enrolled between August 1, 2022, and August 31, 2023. Of these, 26 in the intervention group and 30 in the control group were analyzed, excluding 10 participants who dropped out of the study. The mean baseline HbA_1c_ levels were 8.3% (SD 0.6%) in the intervention group and 8.0% (SD 0.5%) in the control group. At the 3-month follow-up, mean HbA_1c_ decreased by 0.5%-7.8% (SD 0.9%; *P*=.01) in the intervention group and by 0.2%-7.8% (SD 0.7%) in the control group. HbA_1c_ levels substantially improved during the first 3 months with both face-to-face education and remote glucose monitoring. However, HbA_1c_ tended to increase during the 3- to 6-month follow-up in the intervention group without the remote monitoring service. Satisfaction with diabetes treatment significantly improved at the end of the study compared with baseline in the intervention group (mean change +3.6 points; *P*=.006). Medication adherence improved in both groups, with no significant difference at 6 months (*P*=.59), although the intervention group showed a greater increase from baseline. Subgroup analysis indicated that the reduction in HbA_1c_ was greater for patients with baseline HbA_1c_ levels ≥8.0%, those aged ≥65 years, smokers, drinkers, and those with obesity in the intervention group.

**Conclusions:**

The digital education platform for personalized diabetes management may be beneficial for glycemic control in type 2 diabetes mellitus. Its effectiveness appears to be enhanced when physicians provide personalized face-to-face education combined with remote feedback.

**Trial Registration:**

Clinical Research Information Service (CRiS) of Republic of Korea KCT0007953; https://cris.nih.go.kr/cris/search/detailSearch.do?seq=23507&search_page=L

## Introduction

Diabetes is a global health challenge, affecting 540 million people worldwide as of 2021—a number projected to reach 783 million by 2045 [1]. In Korea, the prevalence of diabetes has steadily increased over the past 9 years, reaching 16.7% in 2020; however, only 25% of these individuals achieved a glycated hemoglobin (HbA_1c_) level of <6.5% [2].

Effective diabetes management is crucial, as patients with diabetes are responsible for making most of their own health decisions, including dietary choices, physical activity, blood glucose monitoring, carbohydrate counting, and insulin dosage adjustments [3]. Recognizing the complexity of diabetes management, the American Diabetes Association (ADA) guidelines emphasize “diabetes self-management education and support” (DSMES) as an essential component of comprehensive diabetes treatment [4]. Studies conducted in the United Kingdom, such as the patient-centered, group-based self-management program (X-PERT) and the “Diabetes Education and Self-Management for Ongoing and Newly Diagnosed” (DESMOND) program [5,6], along with the “Structured Intensive Diabetes Education Program” (SIDEP) study in Korea [7], have demonstrated that DSMES significantly improves glycemic control. Additionally, DSMES has been shown to reduce HbA_1c_ levels, enhance quality of life, and decrease hospitalizations and health care costs [3,8].

Patients with type 2 diabetes mellitus (T2DM) present with diverse characteristics, including age, sex, BMI, and lifestyle habits. These factors—along with differences in insulin resistance, insulin secretion capacity, and the presence of comorbidities—contribute to the heterogeneity of diabetes and its related complications [9-12]. Therefore, it is crucial to provide individualized education tailored to patient-specific characteristics rather than relying solely on general education.

Given the chronic nature of diabetes, maintaining consistent blood glucose control over the long term is particularly challenging [13]. Therefore, in addition to encouraging patient self-monitoring, effective diabetes management requires medical professionals to provide continuous, appropriate, and patient-specific feedback through education. Considering patients’ diverse characteristics and the need for ongoing professional oversight, it is essential that physicians—who best understand their patients—deliver in-person education tailored to individual needs. Moreover, providing blood glucose monitoring to patients may enhance diabetes management.

Recent advancements in information technology have led to the development of various digital health care systems. In line with these advancements, the ADA now recommends that DSMES can be effectively delivered through telehealth and digital health care platforms [4]. Studies have explored glucose control via apps, web-based platforms, and message-based diabetes education programs [14-16]. However, these studies primarily focus on patients who measure their own blood glucose levels and receive standardized feedback, falling short of achieving individualized treatment and management. More effective diabetes treatment and management can be achieved when patients monitor their blood glucose levels under physician guidance and receive immediate, appropriate feedback from their physicians.

In response to this need, a digital-based smart education platform for physicians and patients, called Doctorvice (iKooB Inc.), has been developed [17]. This system provides a comprehensive web interface, Doctorvice Clinic, enabling physicians to quickly access patients’ clinical information and deliver educational messages. Physicians can conduct face-to-face education by selecting appropriate content from a web-based library containing over 3000 pages of educational materials or by customizing additional instructions on a digital canvas. The educational content can be transmitted to patients via SMS text messages or the Doctorvice app, allowing them to receive and review the materials repeatedly. Patients can upload self-measured blood glucose (SMBG) data through the app, which physicians can access via the web at any time to provide additional educational content on topics such as healthy eating habits, physical activity, and diabetes self-care. This bidirectional communication supports an educational approach that combines face-to-face sessions with continuous remote monitoring and feedback.

In this study, we aimed to evaluate the effectiveness of a physician-led digital education platform on glycemic control in patients with T2DM. Specifically, we hypothesized that patients receiving both face-to-face education and continuous remote monitoring and feedback from physicians would show greater improvements in glycemic control compared with those receiving only face-to-face education during outpatient visits, without subsequent feedback.

## Methods

### Study Aim

This study was a 6-month, nonblinded randomized controlled trial (KCT0007953) involving patients with T2DM who visited the Diabetes Clinic at Seoul St. Mary’s Hospital between August 1, 2022, and August 31, 2023. The study utilized a digital education platform to assess its effect on the diabetes care model, where physicians provided individualized face-to-face education based on digital content. Patients uploaded their blood glucose levels and received remote feedback from the physician. Moreover, we examined the difference between providing face-to-face education with continuous remote feedback and providing face-to-face education alone.

### Study Design

#### Participants

Patients with T2DM aged ≥19 years and with an HbA_1c_ level of 7.5%-9.5% were enrolled. Exclusion criteria were patients with type 1 diabetes mellitus, latent autoimmune diabetes in adults, gestational diabetes, or those using insulin. Additionally, patients who required immediate treatment for acute diabetic complications, had serious medical conditions, were infected, underwent surgery, or had a serious injury were excluded. Further, individuals with cognitive decline, illiteracy, or no access to a smartphone were excluded from the study due to difficulties using the digital smart platform. People with no prior history of accessing the app were classified as nonusers and excluded.

#### Intervention and Control Groups

The participants were randomly assigned to the intervention and control groups in a 1:1 ratio using a random allocation table created by the Clinical Trial Support Center. Both groups received basic education on SMBG, blood pressure (BP) measurement, and lifestyle modification regimens in accordance with the clinical practice guidelines of the Korean Diabetes Association [2]. Outpatient visits were conducted at 3-month intervals over a 6-month period, with questionnaires administered at the beginning and end of the study, and blood tests performed at 0, 3, and 6 months. The blood tests included measurements of blood glucose, HbA_1c_, blood urea nitrogen, creatinine, liver function tests (aspartate aminotransferase and alanine aminotransferase), and lipid profiles (total cholesterol, triglycerides, high-density lipoprotein cholesterol, and low-density lipoprotein cholesterol). BP, weight, and height were assessed during each outpatient visit.

Alcohol consumption was categorized into 2 groups: nondrinkers and drinkers. Nondrinkers were defined as individuals who did not consume alcohol, while drinkers were those who consumed alcohol, regardless of the amount. Obesity was classified into 5 stages based on BMI values: underweight (<18.5 kg/m^2^), normal weight (18.5-22.9 kg/m^2^), overweight (23.0-24.9 kg/m^2^), obesity class I (25.0-29.9 kg/m^2^), and obesity class II (≥30 kg/m^2^).

#### Control Group—Usual Care Group

The control group received standard face-to-face care at the clinic, with diabetes education provided at registration, at the 3-month follow-up, and at the end of the study. Participants were trained on SMBG levels at enrollment and were asked to record these levels in their own notebooks. They did not use the digital education platform, Doctorvice, and therefore did not receive feedback from the physician.

#### Intervention Group—The Digital Education Platform User Group

In the intervention group, we aimed to assess the effects of a management model that integrates digital content–based education delivered through a platform during face-to-face care at outpatient clinics, combined with remote monitoring and feedback provided by the physician at home. Digital content–based education was provided at the time of patient registration, and the educational content was delivered through the patient’s app (Doctorvice) or via SMS text messages. For the first 3 months, patients uploaded their SMBG data via the app, and the physician regularly provided appropriate feedback, including messages and content. Feedback was given at 2-3-week intervals, depending on the patient’s SMBG levels. The feedback included newly created educational content tailored to each patient based on their physician’s recommendations, covering topics such as diet control, physical activity, and diabetes self-care.

**Figure 1:**
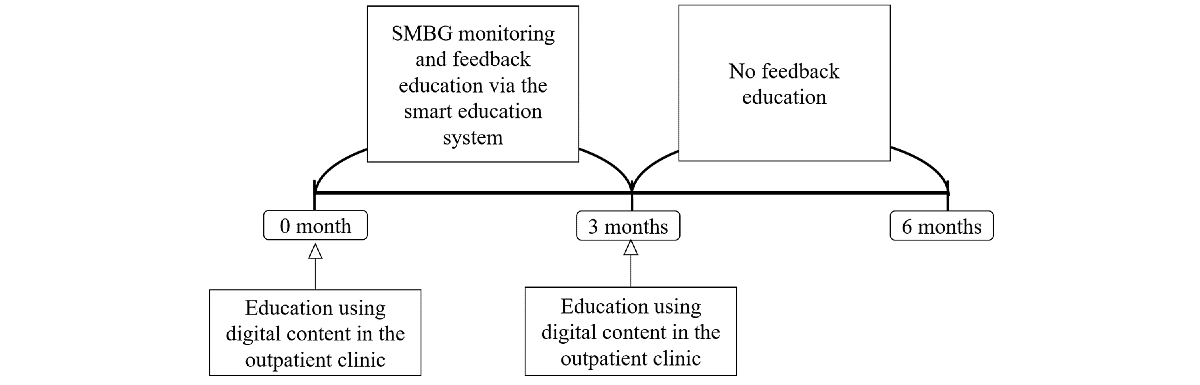
Study design in the intervention group. SMBG: self-measured blood glucose.

The platform allowed physicians to view patients’ basic information at a glance, including BMI, medical history, family history, and alcohol and smoking history, as well as SMBG data, which facilitated personalized education. From months 3 to 6 (end of the study), patients were instructed to measure their own blood glucose levels and upload the values via the app; however, physicians were not allowed to provide feedback during this period. Thus, only self-management was maintained from months 3 to 6 (Figure 1). By structuring the study in this manner, we were able to evaluate the effects of remote education.

### Digital Education Platform

The digital education platform (Doctorvice) serves as a medical consulting and support system that connects physicians and patients. It includes Doctorvice Clinic, a web-based platform accessible to physicians during outpatient clinic visits, and the Doctorvice app, which patients can use on their mobile devices. Physicians can use Doctorvice Clinic to review a patient’s clinical information (eg, age, sex, medical history, family history, and smoking and alcohol history) at a glance, as well as monitor blood glucose and BP data uploaded by the patient (see Figure S1 in Multimedia Appendix 1).

The platform includes a digital content library containing approximately 3000 pieces of content related to diabetes and hypertension education. The content is categorized by disease and symptoms, diagnosis, treatment, side effects, medications, complications, prevention, and management. Physicians can use these contents to provide personalized education based on each patient’s characteristics. Additionally, they can utilize the web canvas for further customization. Physicians can select content relevant to the patient’s specific situation (eg, food intake, exercise, moderation in alcohol consumption, smoking cessation, and medication adherence) and place it on the canvas. They can also edit the content on the canvas by drawing pictures or writing text using the editing tool (see Figure S2 in Multimedia Appendix 1). This allows the creation of personalized educational content tailored to the patient, which can be sent via SMS text messages or the Doctorvice app. Patients can then access and review this content repeatedly on their smartphones.

Patients can upload their SMBG or BP data through the Doctorvice app (see Figure S3 in Multimedia Appendix 1). Physicians can monitor these uploaded data in real time via Doctorvice Clinic and provide feedback through messages and personalized content at any time. This platform fosters a continuous connection between physicians and patients by sharing content and data, facilitating both face-to-face and remote interactions as needed.

### Questionnaires

This study selected 3 questionnaires to assess the clinical impact of the smart education platform in strengthening diabetes self-management behavior and measuring patient management patterns. The questionnaires included satisfaction with diabetes treatment, diabetes stress measurement, and adherence to diabetes medication (see Tables S1-S3 in Multimedia Appendix 1) [18-20]. Satisfaction with diabetes treatment was measured using 8 questions, with a maximum score of 48 points, where higher scores indicate greater satisfaction. Diabetes stress was assessed with 17 questions, with a maximum score of 85 points; higher scores reflect higher stress levels. Adherence to diabetes medication was evaluated with 8 questions, where a “no” answer received 1 point. The final question, “How often do you have difficulty remembering to take all your diabetes medications?” was scored from 4 (never) to 0 (always), for a total possible score of 11 points. Higher scores indicated greater adherence. These 3 surveys were administered at enrollment and again at the end of the study (6 months later) by the same research nurse to minimize investigator bias.

### Outcomes

The primary outcome measure was the difference in HbA_1c_ levels between the control and intervention groups. Changes in HbA_1c_ levels from baseline to 3 months and from 3 to 6 months were analyzed. Subgroup analyses were performed based on SMBG upload frequency, age, baseline HbA_1c_ level, smoking status, alcohol consumption, and BMI. The secondary outcome was the difference in questionnaire results before and after the study.

### Statistical Analysis

Statistical analyses were performed using SPSS (version 27.0; IBM Corp.) and Rex-Pro (version 3.6.0; Rexsoft, Co. Ltd.). The Kolmogorov-Smirnov test or the Q-Q plot was used to assess the normality of the data. Descriptive statistics are presented as means and SDs for continuous variables, and as frequencies with proportions for categorical variables. Two-tailed independent sample paired *t* tests and ANOVA were used to compare differences between groups for normally and nonnormally distributed data, respectively. Paired *t* tests and Wilcoxon tests were used to evaluate pre- and postintervention differences for normally and nonnormally distributed data, respectively. The Pearson correlation coefficient was applied to assess relationships between variables.

### Ethical Considerations

This study was approved by the institutional review board at The Catholic University of Korea (approval number KC19FESI0604) in September 2019. Informed consent was obtained from all participants. To protect their privacy, an independent anonymization officer, who was not involved in the study intervention, was assigned to handle the anonymization process. No monetary compensation was provided to the participants.

## Results

### Baseline Characteristics

**Figure 2:**
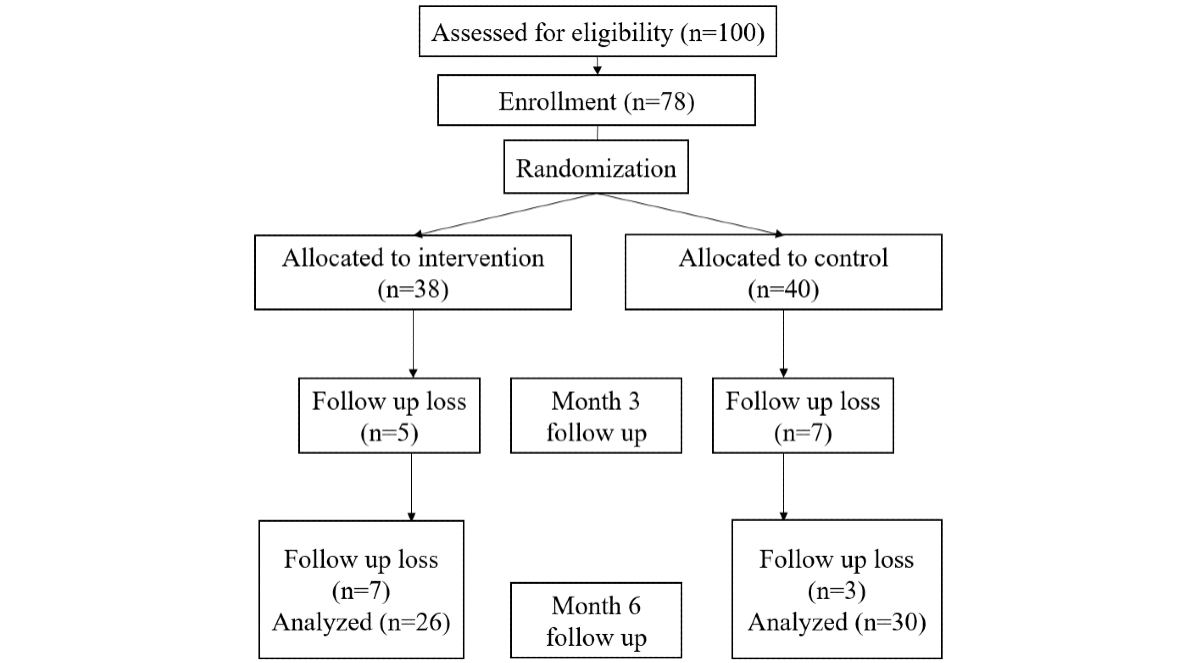
CONSORT flowchart.

A total of 66 participants were initially enrolled in the study. After excluding 10 individuals who did not attend the outpatient clinic at 3 or 6 months, the final analysis included 26 (46%) participants in the intervention group and 30 (54%) participants in the control group. The mean age at baseline for these 56 participants was 60.3 years, and 42 (75%) were male (Table 1). The mean HbA_1c_ levels were 8.1% (SD 0.5%) overall, 8.3% (SD 0.6%) in the intervention group, and 8.0% (SD 0.5%) in the control group. The average BMI was 26.5 (SD 4.6) kg/m^2^ in the intervention group and 25.5 (SD 3.4) kg/m^2^ in the control group. There were no significant differences in the proportions of nonsmokers, ex-smokers, and current smokers (*P*=.28), nor in alcohol consumption (*P*=.28). The average number of diabetes medications was 2.9 (SD 0.7) in the intervention group and 2.8 (SD 0.8) in the control group. Of the 56 participants, 47 completed the surveys at both the beginning and end of the study, including 20 from the intervention group and 27 from the control group. Figure 2 presents the CONSORT (Consolidated Standards of Reporting Trials) flowchart (also see Multimedia Appendix 2 for the CONSORT checklist).

**Table 1:** Baseline clinical characteristics of the study participants.

Characteristics			Total (N=56)		Intervention group (n=26)		Control group (n=30)		*P* value
Age (years), mean (SD)			60.3 (9.1)		59.0 (10.8)		61.3 (7.3)		.37
Male sex, n (%)			42 (75)		22 (85)		20 (67)		.22
Glucose (mg/dL), mean (SD)			154 (30)		159 (34)		150 (25)		.26
Glycated hemoglobin (HbA_1c_^a^; %), mean (SD)			8.1 (0.5)		8.3 (0.6)		8.0 (0.5)		.10
Diabetes mellitus duration (years), mean (SD)			12.4 (7.0)		12.4 (7.5)		12.5 (6.6)		.94
Systolic blood pressure (mmHg), mean (SD)			124 (13)		126 (7)		123 (16)		.47
Diastolic blood pressure (mmHg), mean (SD)			73 (11)		76 (10)		70 (12)		.046
Urea nitrogen (mg/dL), mean (SD)			18.1 (5.5)		18.0 (6.2)		18.1 (4.9)		.95
Creatinine (mg/dL), mean (SD)			0.89 (0.27)		0.95 (0.30)		0.84 (0.25)		.15
Estimated glomerular filtration rate (mL/min/1.73 m^2^), mean (SD)			87.6 (18.7)		86.1 (20.7)		88.8 (16.4)		.59
Aspartate aminotransferase (U/L), mean (SD)			27 (13)		30 (14)		24 (11)		.07
Alanine aminotransferase (U/L), mean (SD)			39 (57)		33 (19)		43 (77)		.48
Gamma-glutamyl transferase (U/L), mean (SD)			56 (110)		62 (88)		51 (129)		.70
Total cholesterol (mg/dL), mean (SD)			142 (31)		144 (34)		140 (28)		.62
Triglycerides (mg/dL), mean (SD)			139 (78)		156 (87)		124 (68)		.15
High-density lipoprotein cholesterol (mg/dL), mean (SD)			45 (11)		46 (12)		45 (10)		.60
Low-density lipoprotein cholesterol (mg/dL), mean (SD)			70 (24)		71 (25)		70 (25)		.88
Body weight (kg), mean (SD)			72.1 (14.5)		74.5 (17.3)		70.1 (11.5)		.28
BMI (kg/m^2^), mean (SD)			26.0 (4.0)		26.5 (4.6)		25.5 (3.4)		.37
Number of oral hypoglycemic agents, mean (SD)			2.8 (0.7)		2.9 (0.7)		2.8 (0.8)		.95
**Smoking, n (%)**									.28
	None	25 (44.6)		9 (35)		16 (53)			
	Ex	22 (39.3)		13 (50)		9 (30)			
	Current	9 (16.1)		4 (15)		5 (17)			
Alcohol consumption, n (%)			29 (52)		16 (62)		13 (43)		.73
Total education time (minutes), mean (SD)			N/A^b^		22.6 (9.7)		N/A		N/A
Number of education sessions, mean (SD)			N/A		4.4 (1.1)		N/A		N/A
Education time per session (minutes), mean (SD)			N/A		5.2 (2.1)		N/A		N/A
Number of education contents sent to patients, mean (SD)			N/A		24.8 (7.8)		N/A		N/A

^a^HbA_1c_: glycated hemoglobin.

^b^N/A: not applicable.

In the intervention group, the average duration of each educational session using the platform was 22.6 (SD 9.7) minutes, with an average of 4.4 (SD 1.1) sessions held over the first 3 months. Additionally, a mean of 24.8 (SD 7.8) pieces of educational content was sent to participants during this period, resulting in an average of 5.2 minutes of education per participant.

Although it was not possible to precisely measure the education time in the control group, given the unique characteristics of the outpatient care environment in Korea—where the average consultation time per patient typically ranges from 3 to 5 minutes—it can be assumed that the education time in the control group was similar to this range.

### Change in HbA^1c^

#### Changes in HbA^1c^ Over the First 3 Months

**Figure 3:**
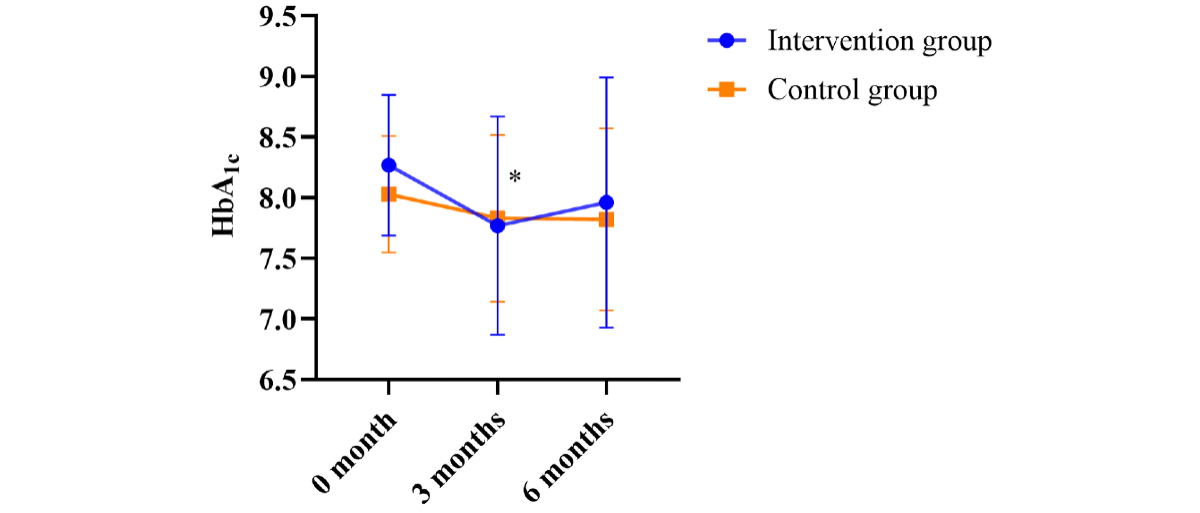
Changes in glycated hemoglobin (HbA_1c_) after education using the digital platform. *The change in HbA_1c_ from baseline to 3 months was significant in the intervention group (*P*=.01).

During the first 3 months, the intervention group experienced a significant reduction in HbA_1c_ levels, decreasing from a mean baseline level of 8.3% (SD 0.6%) to 7.8% (SD 0.9%), a change of –0.5% (*P*=.01; Figure 3). By contrast, the control group showed a nonsignificant decrease in mean HbA_1c_ levels, from 8.0% (SD 0.5%) to 7.8% (SD 0.7%), a change of –0.2% (*P*=.13).

#### Changes in HbA^1c^ Between 3 and 6 Months

In the intervention group, HbA_1c_ levels showed a slight increase of 0.2% (from a mean of 7.8%, SD 0.9%, at 3 months to 8.0%, SD 1.0%, at 6 months), although this change was not statistically significant (*P*=.28). By contrast, the control group exhibited minimal change, with no significant difference in HbA_1c_ levels, remaining at a mean of 7.8% (SD 0.7%) at 3 months and a mean of 7.8% (SD 0.8%) at 6 months (*P*=.92).

#### Changes in HbA^1c^ Over 6 Months

At the 6-month follow-up, the HbA_1c_ levels in the intervention group decreased by 0.3% from baseline (*P*=.12), while in the control group, they decreased by 0.2% (*P*=.18). However, neither group showed a statistically significant reduction compared with baseline.

### Analysis of Questionnaires

#### Satisfaction With Diabetes Treatment

The baseline Cronbach α for both the intervention and control groups was 0.8, indicating a high level of reliability. At the 6-month follow-up, the Cronbach α decreased to 0.6 for both groups, maintaining a moderate level of reliability.

**Figure 4:**
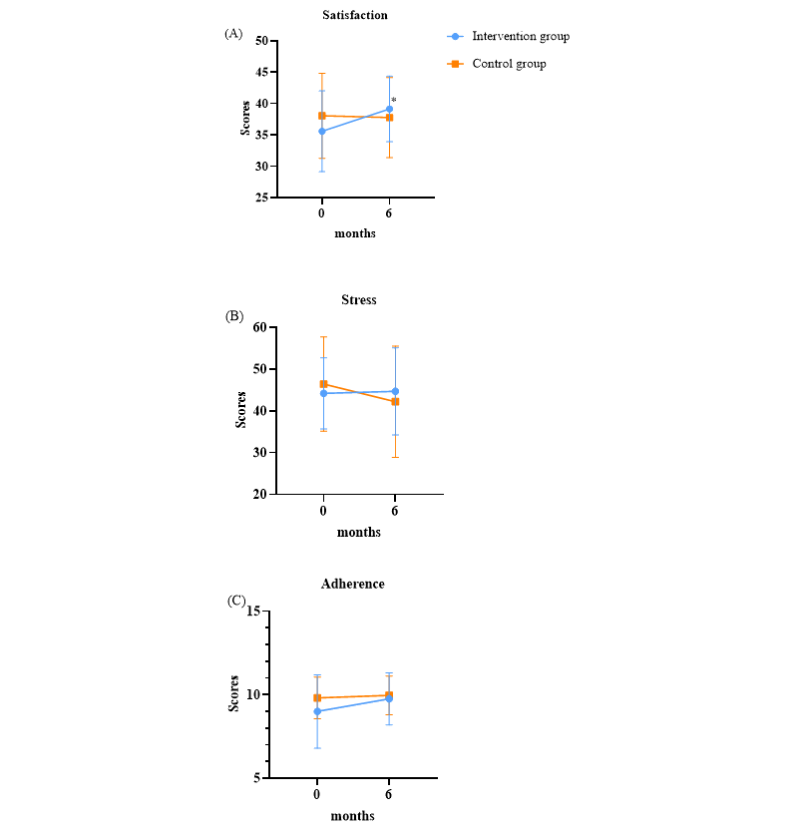
(A) Changes in the “satisfaction with diabetes treatment” score. (B) Changes in the “diabetes stress measurement” score. (C) Changes in the “adherence of diabetes medication” score. *The change in the satisfaction score was significant in the intervention group (*P*=.006). Vertical error bars indicate SD.

The mean baseline satisfaction score for diabetes treatment was 35.6 (SD 6.4) points in the intervention group and 38.1 (SD 6.8) points in the control group (Figure 4A). At 6 months, the intervention group showed a significant increase of 3.6 points, reaching a mean of 39.2 (SD 5.2) points, while the control group experienced a slight decrease of 0.3 points, reaching a mean of 37.8 (SD 6.4) points. The intervention group demonstrated a significant improvement in satisfaction with diabetes treatment compared with the baseline (*P*=.006).

#### Diabetes Stress Measurement

The baseline Cronbach α was 0.7 for the intervention group and 0.9 for the control group, indicating a high level of reliability. At the 6-month follow-up, the Cronbach α increased to 0.8 in the intervention group, while it remained at 0.9 in the control group, maintaining a high level of reliability.

The mean baseline diabetes stress score was 44.2 (SD 8.5) points in the intervention group and 46.4 (SD 11.3) points in the control group (Figure 4B). At 6 months, the mean score in the intervention group was 44.7 (SD 10.4) points, while the control group had a mean score of 42.2 (SD 13.3) points. The change in stress scores differed significantly; the intervention group experienced an average change of 0.5 (SD 9.7), whereas the control group showed a decrease of –4.2 (SD 6.8). This indicates that stress levels improved more in the control group compared with the intervention group, with the difference approaching significance (*P*=.06).

#### Adherence to Diabetes Medication

The baseline Cronbach α was 0.6 for the intervention group and 0.4 for the control group. At the 6-month follow-up, Cronbach α decreased to 0.4 in the intervention group and 0.3 in the control group.

The mean baseline adherence score was 9.0 (SD 2.2) points in the intervention group and 9.8 (SD 1.2) points in the control group, with no significant difference between the 2 groups (*P*=.11). However, individuals in the control group exhibited better adherence at the time of enrollment (Figure 4C). At the 6-month follow-up, the intervention group saw an increase of 0.8 points, reaching a mean of 9.8 (SD 1.6) points, while the control group’s score increased by 0.2 points to a mean of 10.0 (SD 1.2) points. There was no significant difference in adherence scores between the 2 groups (*P*=.59), although adherence scores in the intervention group showed a greater tendency for improvement compared with the control group.

### Correlation Analysis Between Questionnaires’ Score and HbA^1c^

Correlation analysis was conducted to explore the relationship between changes in HbA_1c_ levels and the scores from the survey results over the course of the study.

In the intervention group, the change in HbA_1c_ levels during the first 3 months showed a strong and significant (*P*=.17) inverse relationship with the change in HbA_1c_ levels during the 3-6 months (–0.7095), indicating that improvement in HbA_1c_ levels during the first 3 months was associated with deterioration in HbA_1c_ levels during the subsequent 3 months (Table 2). The change in stress scores was associated with the change in HbA_1c_ levels during the first 3 months (–0.3339), the entire 6-month study period (0.4372), and the 3-6-month period (0.6294). This suggests that during the first 3 months, improvement in HbA_1c_ levels was moderately associated with reduced stress. However, improvement in HbA_1c_ levels over the full 6 months and during the 3-6-month period was associated with increased stress. Additionally, although the correlation between HbA_1c_ improvement and adherence scores was not statistically significant (*P*=.17), there was a tendency for better adherence to be associated with improved HbA_1c_ levels in the intervention group during the first 3 months and over the entire 6-month period.

In the control group, the Pearson correlation between the degree of HbA_1c_ improvement over the 6-month period and the degree of stress improvement was 0.4475 (Table 3).

**Table 2:** The Pearson correlation in the intervention group.

Variable	HbA_1c_^a^ 0-3^b^ (*P* value)	HbA_1c_ 0-6^c^ (*P* value)	HbA_1c_ 3-6^d^ (*P* value)	Satisfaction 0-6^e^ (*P* value)	Stress 0-6^f^ (*P* value)	Adherence 0-6^g^ (*P* value)
HbA_1c_ 0-3	—^h^	0.2881 (.21)	–0.7095 (<.001)	–0.0097 (.97)	–0.3339 (.15)	–0.3160 (.17)
HbA_1c_ 0-6	0.2881 (.22)	—	0.4704 (.04)	0.0051 (.98)	0.4372 (.05)	–0.3229 (.17)
HbA_1c_ 3-6	–0.7095 (<.001)	0.4704 (.04)	—	0.0126 (.96)	0.6294 (.003)	0.0536 (.82)
Satisfaction 0-6	–0.0097 (.97)	0.0051 (.98)	0.0126 (.96)	—	–0.3945 (.09)	0.5198 (.02)
Stress 0-6	–0.3339 (.15)	0.4372 (.05)	0.6294 (.003)	–0.3945 (.09)	—	–0.3795 (.10)
Adherence 0-6	–0.3160 (.17)	–0.3229 (.17)	0.0536 (.82)	0.5198(.02)	–0.3795 (.10)	—

^a^HbA_1c_: glycated hemoglobin.

^b^HbA_1c_ 0-3: changes in HbA_1c_ during the first 3 months.

^c^HbA_1c_ 0-6: changes in HbA_1c_ during the first 6 months.

^d^HbA_1c_ 3-6: changes in HbA_1c_ during 3-6 months.

^e^Satisfaction 0-6: changes in satisfaction with diabetes treatment over 6 months.

^f^Stress 0-6: changes in diabetes stress measurement over 6 months.

^g^Adherence 0-6: changes in adherence to diabetes medications over 6 months.

^h^Not applicable.

**Table 3:** The Pearson correlation in the control group.

Variable	HbA_1c_^a^ 0-3^b^ (*P* value)	HbA_1c_ 0-6^c^ (*P* value)	HbA_1c_ 3-6^d^ (*P* value)	Satisfaction 0-6^e^ (*P* value)	Stress 0-6^f^ (*P* value)	Adherence 0-6^g^ (*P* value)
HbA_1c_ 0-3	—^h^	0.7331 (<.001)	–0.1907 (.34)	0.0133 (.95)	0.3083 (.12)	0.0310 (.88)
HbA_1c_ 0-6	0.7331 (<.001)	—	0.5278 (.005)	–0.1690 (.40)	0.4475 (.02)	0.0571 (.78)
HbA_1c_ 3-6	–0.1907 (.34)	0.5278 (.005)	—	–0.2605 (.19)	0.2608 (.19)	0.0437 (.83)
Satisfaction 0-6	0.0133 (.95)	–0.1690 (.40)	–0.2605 (.19)	—	–0.1907 (.34)	0.1357 (.50)
Stress 0-6	0.3083 (.12)	0.4475 (.02)	0.2608 (.19)	–0.1907 (.34)	—	0.0051 (.98)
Adherence 0-6	0.0310 (.88)	0.0571 (.78)	0.0437 (.83)	0.1357 (.50)	0.0051 (.98)	—

^a^HbA_1c_: glycated hemoglobin.

^b^HbA_1c_ 0-3: changes in HbA_1c_ during the first 3 months.

^c^HbA_1c_ 0-6: changes in HbA_1c_ during the first 6 months.

^d^HbA_1c_ 3-6: changes in HbA_1c_ during 3-6 months.

^e^Satisfaction 0-6: changes in satisfaction with diabetes treatment over 6 months.

^f^Stress 0-6: changes in diabetes stress measurement over 6 months.

^g^Adherence 0-6: changes in adherence to diabetes medications over 6 months.

^h^Not applicable.

### Subgroup Analysis

#### Change in HbA^1c^ According to SMBG Upload Frequency

**Figure 5:**
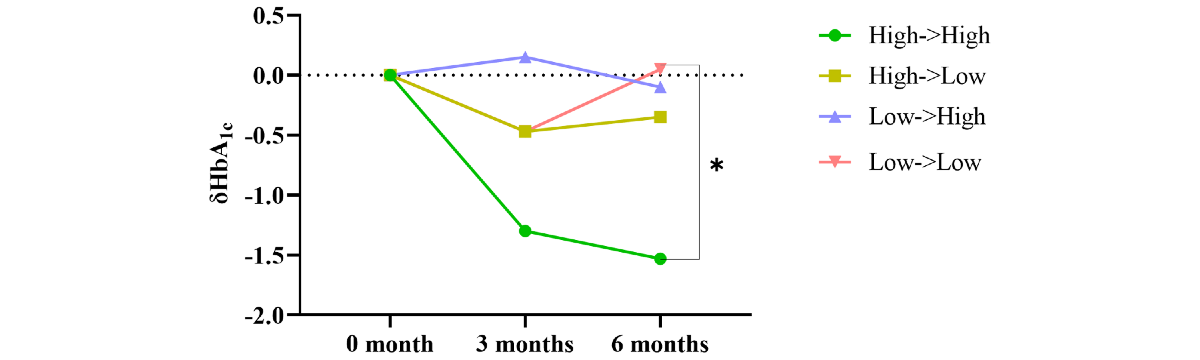
Changes in glycated hemoglobin (HbA_1c_) according to self-measured blood glucose (SMBG) upload frequency. *Group 1 had a significantly greater reduction in HbA_1c_ compared with group 4 (post hoc analysis *P*=.02).

Participants who uploaded their blood glucose levels via the app more than once a week were classified as “high,” whereas those who uploaded less frequently were classified as “low.” The intervention group was subdivided based on their upload frequency in the first and last 3 months: group 1 consisted of participants classified as “high” for both periods, group 2 as “high” for the first 3 months and “low” for the last 3 months, group 3 as “low” for the first 3 months and “high” for the last 3 months, and group 4 as “low” for both periods. In the intervention group, there were 4 patients in group 1, 6 in group 2, 4 in group 3, and 12 in group 4. Compared with baseline, HbA_1c_ levels improved by –1.3% in group 1, –0.5% in group 2, and –0.5% in group 4 at 3 months, while HbA_1c_ increased by 0.2% in group 3. Notably, patients classified as “high” throughout the 6 months (group 1) had a significantly greater reduction in HbA_1c_ compared with those classified as “low” for the entire 6-month period (group 4; post hoc analysis *P*=.02; Table 4, Figure 5). The control group could not be analyzed for this metric due to the absence of SMBG uploads via the app.

**Table 4:** Changes in HbA_1c_^a^ according to SMBG^b^ upload frequency.

Group	Baseline, mean (SD)	3 months, mean (SD)	*P* value	∆^c^ (baseline to 3 months) (SD)	6 months, mean (SD)	*P* value	∆ (3-6 months) (SD)
Group 1 (n=4)	8.4 (0.7)	7.1 (0.9)	.14	–1.3 (1.3)	6.9 (0.8)	.49	–0.2 (0.6)
Group 2 (n=6)	8.4 (0.6)	7.9 (1.3)	.33	–0.5 (1.1)	8.1 (1.2)	.79	0.1 (1.0)
Group 3 (n=4)	8.2 (0.4)	8.3 (0.4)	.64	0.2 (0.6)	8.1 (0.5)	.43	–0.3 (0.5)
Group 4 (n=12)	8.2 (0.6)	7.7 (0.7)	.03	–0.5 (0.7)	8.3 (1.0)	.08	0.5 (0.9)

^a^HbA_1c_: glycated hemoglobin.

^b^SMBG: self-measured blood glucose.

^c^∆: difference in HbA_1c_.

#### Change in HbA^1c^ According to Age

**Figure 6:**
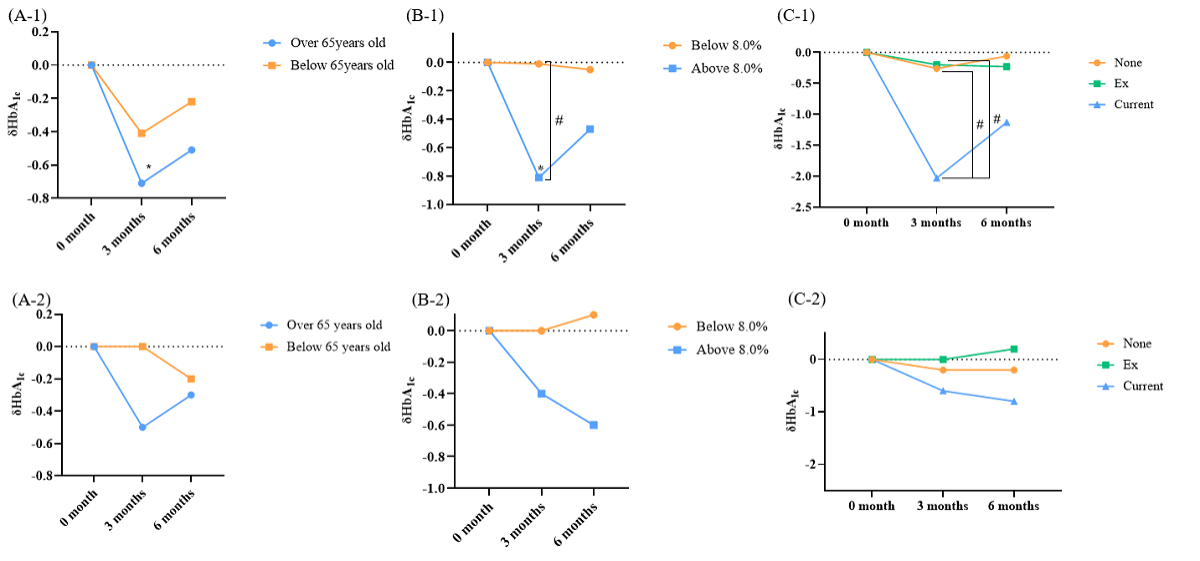
(A-1) Changes in HbA_1c_ according to age (≥65 years vs <65 years) in the intervention group. (A-2) Changes in HbA_1c_ according to age (≥65 years vs <65 years) in the control group. (B-1) Changes in HbA_1c_ according to baseline HbA_1c_ (8.0%) in the intervention group. (B-2) Changes in HbA_1c_ according to baseline HbA_1c_ (8.0%) in the control group. (C-1) According to smoking status in the intervention group. (C-2) Changes in HbA_1c_ according to smoking status in the control group. (D-1) Changes in HbA_1c_ according to drinking status in the intervention group. (D-2) Changes in HbA_1c_ according to drinking status in the control group. (E-1) Changes in HbA_1c_ according to BMI level in the intervention group. (E-2) Changes in HbA_1c_ according to BMI level in the control group. *Compared with baseline, change in HbA_1c_ at 3 months was significant in the intervention group. #There was a significant difference in the degree of HbA_1c_ change between the 2 groups. HbA_1c_: glycated hemoglobin.

The degree of improvement in HbA_1c_ levels was assessed by classifying patients into 2 age groups: those aged <65 years and ≥65 years. In the intervention group, there were 18 individuals aged <65 years, with a mean baseline HbA_1c_ level of 8.1% (SD 0.6%; Figure 6A-1). At 3 months, HbA_1c_ improved by –0.4%, reaching a mean of 7.7% (SD 1.0%; *P*=.10). In the intervention group, there were 8 individuals aged ≥65 years, with a mean baseline HbA_1c_ level of 8.6% (SD 0.5%). At 3 months, their HbA_1c_ improved by –0.7%, reaching a mean of 7.9% (SD 0.7%; *P*=.03). In the control group, there were 19 individuals aged <65 years, with a mean baseline HbA_1c_ level of 8.0% (SD 0.5%). Additionally, there were 11 individuals aged ≥65 years, with a mean baseline HbA_1c_ level of 8.0% (SD 0.6%). There was no significant difference in HbA_1c_ improvement between the age groups in the control group (*P*=.49; Figure 6A-2).

#### Change in HbA^1c^ According to Baseline HbA^1c^

In the intervention group, 16 participants had an HbA_1c_ level ≥8.0%, while 10 participants had an HbA_1c_ level <8.0%. At 3 months, the group with HbA_1c_ ≥8.0% showed an improvement of –0.8% (*P*=.006) compared with baseline, while the group with HbA_1c_ <8.0% showed only a slight change (*P*=.95) compared with baseline. A significant difference was observed between the 2 groups (*P*=.03; Figure 6B-1). In the control group, 13 patients had an HbA_1c_ level ≥8.0%, while 17 patients had an HbA_1c_ level <8.0%. No significant difference was observed between the 2 groups (*P*=.30; Figure 6B-2).

#### Change in HbA^1c^ According to the Smoking Status

In the intervention group, 9 participants were nonsmokers, 13 were ex-smokers, and 4 were current smokers. At 3 months, the mean improvement in HbA_1c_ compared with baseline was –0.3% (SD 0.5%) in nonsmokers, –0.2% (SD 0.6%) in ex-smokers, and –2.0% (SD 1.0%) in current smokers (Figure 6C-1). Post hoc analysis revealed significant differences between current smokers and both nonsmokers (*P*<.001) and ex-smokers (*P*<.001), but no significant difference between nonsmokers and ex-smokers (*P*=.98)

In the control group, 16 participants were nonsmokers, 9 were ex-smokers, and 5 were current smokers. Changes in HbA_1c_ did not differ significantly among the smoking status groups (post hoc analysis: noncurrent, *P*=.52; ex-current, *P*=.30; and non-ex, *P*=.76; Figure 6C-2).

#### Change in HbA^1c^ According to Alcohol Consumption

In the intervention group, there were 10 nondrinkers and 16 alcohol drinkers. At 3 months, the improvement in HbA_1c_ compared with baseline was –0.1% in nondrinkers and –0.8% in alcohol drinkers (*P*=.06; Figure 6D-1). The control group included 17 nondrinkers and 13 drinkers. No significant difference was observed between the 2 groups (*P*=.51; Figure 6D-2).

#### Change in HbA^1c^ According to the Baseline BMI Level

Based on baseline BMI, participants were classified into 4 groups: group 1 (normal weight), group 2 (preobesity), group 3 (obesity stage 1), and group 4 (obesity stages 2 and 3), with no participants classified as underweight in any group. In the intervention group, there were 6 participants in group 1, 5 in group 2, 11 in group 3, and 4 in group 4. At 3 months, HbA_1c_ improvement was more pronounced in group 4 compared with the other groups, though this difference was not statistically significant (post hoc analysis: groups 1-2, *P*=.66; groups 1-3, *P*=.80; groups 1-4, *P*=.35; groups 2-3, *P*=.97; groups 2-4, *P*=.06; and groups, 3-4, *P*=.06; Figure 6E-1). In the control group, with 7 participants in group 1, 10 in group 2, 10 in group 3, and 3 in group 4, no significant differences in HbA_1c_ improvement were observed among the groups (*P*=.37; Figure 6E-2).

## Discussion

In this pilot randomized controlled trial involving patients with T2DM, a digital education platform combining face-to-face education with remote feedback over a 3-month period resulted in a significantly greater reduction in HbA_1c_ (–0.5%) compared with conventional education (–0.2%). However, HbA_1c_ levels in the intervention group slightly increased after remote feedback ended. Treatment satisfaction improved significantly in the intervention group, while stress decreased more in the control group. Medication adherence improved modestly in both groups, with no significant between-group differences. Greater HbA_1c_ improvement was associated with more frequent SMBG uploads and was more pronounced in participants with higher baseline HbA_1c_, age ≥65 years, smoking, drinking, or obesity.

This study demonstrated that the digital education platform for personalized diabetes management was beneficial for glycemic control in T2DM and was fairly effective when physicians provided both personalized face-to-face education and remote monitoring and feedback. A significant decrease in HbA_1c_ was observed during the first 3 months, when face-to-face education combined with remote monitoring and feedback was provided through this platform. However, during the 3-6-month period, when education was limited to face-to-face sessions at the outpatient clinic without further feedback from physicians, HbA_1c_ levels tended to increase slightly. Even in the control group, which received only regular face-to-face education at the outpatient clinic, HbA_1c_ levels decreased over the first 3 months, suggesting that initial education may be effective in diabetes management. Nevertheless, the more significant reduction in HbA_1c_ levels observed in the intervention group can be attributed to the additional effect of SMBG uploads and feedback from physicians. The effectiveness of education was enhanced for the intervention group by enabling more continuous learning, as they received digital education and educational materials via SMS text messages or the Doctorvice app. This contributed to a more significant reduction in HbA_1c_ levels. One study has noted that fostering user engagement is essential for diabetes self-management apps to effectively complement clinical care [21]. Another study emphasized that outcomes in diabetes management varied depending on the level of message-based interaction [22]. These findings align with the results of our study, underscoring the importance of patient-centered, personalized, and sustained interaction for optimal diabetes care. The slight increase in HbA_1c_ levels following the discontinuation of remote feedback in our intervention group further reinforces the critical role of ongoing feedback in supporting long-term glycemic control.

In this study, at 3 months, the difference in HbA_1c_ levels between the 2 groups was approximately –0.3%, suggesting that the additional education on diabetes management provided during this period produced results comparable to those of other studies. In a systematic review and meta-analysis of diabetes management using mobile phone apps [23], the difference in HbA_1c_ between the intervention and control groups ranged from –0.69% to –0.28%. Additionally, another meta-analysis indicated that improvements in HbA_1c_ levels occurred only when clinical interventions, such as medication adjustments, were included [24]. Although no medication adjustments were made during this study, the observed improvement in HbA_1c_ levels aligns with findings from other studies that emphasize the importance of physician involvement in diabetes management. In the intervention group, HbA_1c_ levels tended to increase again during the 3- to 6-month period, when remote feedback was no longer provided. This trend aligns with findings from other studies [25,26], which reported the greatest effects during short-term study periods. Furthermore, these studies suggested that the most significant improvements are typically observed around 6 months. The decline in educational effect observed after discontinuing the intervention highlights the importance of sustained support. These findings suggest that health care providers should offer long-term remote education to maintain and reinforce behavioral changes in diabetes care.

Survey results indicated that satisfaction with diabetes treatment and adherence to diabetes medications tended to improve in the intervention group compared with the control group. The non–face-to-face education provided outside of the outpatient clinic served as an additional treatment component, tailored to each patient, which likely contributed to these improvements. However, the reliability of the diabetes medication adherence survey was low, which may be attributed to the small number of items. Additionally, the use of a dichotomous response format (yes/no) may have contributed to the lower reliability. In the diabetes stress measurement, stress scores significantly improved in the control group. The Pearson correlation analysis revealed a negative correlation between changes in HbA_1c_ levels during the first 3 months and stress in the intervention group. The diabetes stress measurement included items on the emotional burden, physician-related distress, regimen-related distress, and diabetes-related interpersonal distress. Although analyzing the changes in scores for each category would have been beneficial, this was not conducted. In the intervention group, the increase in stress may have been influenced by continuous contact, which could have heightened the emotional burden and exacerbated regimen-related distress due to the discrepancy between personal lifestyle and educational content. By contrast, the control group may have perceived the overall experience more positively. Nevertheless, improvements in treatment satisfaction and adherence suggest that the digital intervention still provided meaningful benefits, consistent with previous studies reporting positive effects of digital health interventions on the quality of life in patients with type 2 diabetes [27,28]. A more detailed analysis of individual item scores could have offered a clearer explanation of these findings.

SMBG upload frequency, inferred from blood glucose level uploads via the app, indicated that participants who consistently uploaded their SMBG data over 6 months experienced better HbA_1c_ improvement compared with those with infrequent uploads.

In addition, the effect of the digital education platform on reducing HbA_1c_ levels was notably greater for individuals with HbA_1c_ levels ≥8.0% and those aged ≥65 years. However, some studies suggest that the effect of digital education is more pronounced in patients with higher baseline HbA_1c_ levels, younger age, and shorter disease duration [29], while others indicate that it is effective in older adults regardless of baseline HbA_1c_ levels [30]. The optimal age group for the effect of the digital education platform has not yet been clearly established. Our research suggests that simultaneous face-to-face education and remote feedback can enhance effectiveness, even in older adults. As widely recognized, higher baseline HbA_1c_ levels are associated with greater educational effects. Similarly, our study demonstrated that the effectiveness of the digital education platform was more pronounced in individuals with higher baseline HbA_1c_ levels.

Interestingly, current smokers in the intervention group experienced a more significant reduction in HbA_1c_ levels compared with nonsmokers and ex-smokers. Additionally, there was a trend toward decreased HbA_1c_ levels in alcohol drinkers compared with nondrinkers. Furthermore, the HbA_1c_ reduction in the intervention group was more pronounced among those with a BMI ≥30 kg/m^2^, suggesting that the digital education system may have been particularly effective in encouraging lifestyle improvements through continuous education, especially for individuals with less healthy habits.

The strength of our study lies in the additional survey conducted in the outpatient clinic, which allowed us to confirm the use of the digital education system and assess patients’ health status, behaviors, and adherence from multiple perspectives. This comprehensive approach facilitates a thorough analysis of patient self-management. Unlike previous studies, which did not establish the effect of diabetes self-management through digital education on smoking or other lifestyle groups, our study conducted detailed subgroup analyses to identify specific groups where digital education could be effective. As the age group of smartphone users gradually expands, it is crucial for seniors to actively engage with digital health care. Notably, our study included participants aged ≥65 years, addressing a gap in the existing research. The main strength of this study lies in its attempt to examine the effectiveness of the digital education system across various factors, such as baseline HbA_1c_, age, and lifestyle habits.

However, our study had limitations. The sample size was smaller than initially targeted, with fewer than 30 participants in each group after accounting for dropouts. This small sample size limited the ability to conduct more detailed subgroup analyses and reduced the statistical power of our findings. Additionally, because the patient intervention period was only 6 months—relatively short for evaluating changes in lifestyle habits—only blood glucose control was assessed in diabetes management. Besides, the exact number of SMBGs performed by the control group was unknown, complicating the analysis of improvements in blood glucose control. Another limitation was the insufficient assessment of the digital education platform itself; collecting feedback on satisfaction, needs, and evaluation of the educational content through surveys would have been beneficial for system development. Conducting a survey in the third month would have allowed for a more accurate comparison of the platform’s functions and survey results. Additionally, it would have been beneficial to include not only HbA_1c_ and the 3 survey results but also lifestyle improvements. Further analysis of changes in individual items within each questionnaire would have provided a more detailed understanding of the results. Moreover, analyzing the data according to the categories of educational content would have strengthened the findings.

In conclusion, personalized diabetes management using a digital education system appears to be beneficial for glycemic control in patients with T2DM. The system was particularly effective for individuals who had not achieved optimal glycemic control, were older, or had poorer lifestyle habits. Future research should focus on evaluating the efficacy of this digital education platform in individuals with diverse clinical profiles and conducting longer-term follow-up studies with larger participant groups.

## Appendix

Multimedia Appendix 1 Supplementary tables and figures.

Multimedia Appendix 2 CONSORT-eHEALTH checklist (V 1.6.1).

## Abbreviations

ADA: The American Diabetes Association
BP: blood pressure
CONSORT: Consolidated Standards of Reporting Trials
DESMOND: Diabetes Education and Self-Management for Ongoing and Newly Diagnosed
DSMES: diabetes self-management education and support
HbA_1c_: glycated hemoglobin
SIDEP: Structured Intensive Diabetes Education Program
SMBG: self-measured blood glucose
T2DM: type 2 diabetes mellitus

## Data Availability

The datasets generated or analyzed during this study are available from the corresponding author on reasonable request.
