# Supplementary material for: Clinical Impact of Personalized Physician’s Education and Remote Feedback Via a Digital Platform on Glycemic Control: Pilot Randomized Controlled Trial
Source: JMIR Mhealth Uhealth. 2025 May 1;13:e67151. doi: 10.2196/67151 (PMC12082059; doi:10.2196/67151)

**Supplementary Tables**

**Table S1. Satisfaction with Diabetes Treatment**

| How satisfied are you with your current treatment? | Very satisfied | 6 | 5 | 4 | 3 | 2 | 1 | 0 | Very unsatisfied |
| --- | --- | --- | --- | --- | --- | --- | --- | --- | --- |
| How often have you recently noticed that your blood sugar level has risen to an unacceptable level? | Never | 6 | 5 | 4 | 3 | 2 | 1 | 0 | Mostly noticed |
| How often have you recently noticed that your blood sugar has dropped to an unacceptable level? | Never | 6 | 5 | 4 | 3 | 2 | 1 | 0 | Mostly noticed |
| How convenient do you feel about the treatment you recently received? | Very convenient | 6 | 5 | 4 | 3 | 2 | 1 | 0 | Very inconvenient |
| How well did you find that the treatment you recently received fits into your daily life? | Very well suitable | 6 | 5 | 4 | 3 | 2 | 1 | 0 | Very unsuitable |
| How well do you understand your diabetes? | Very satisfied | 6 | 5 | 4 | 3 | 2 | 1 | 0 | Very unsatisfied |
| Would you recommend this type of treatment? | Definitely recommend | 6 | 5 | 4 | 3 | 2 | 1 | 0 | Never recommend |
| How satisfied are you with continuing to receive your current treatment? | Very satisfied | 6 | 5 | 4 | 3 | 2 | 1 | 0 | Very unsatisfied |

**Table S2. Diabetes Stress Measurement**

| **Diabetes Stress Measurement** | Always | Often | Sometimes | Rare | Never |
| --- | --- | --- | --- | --- | --- |
| Diabetes drains so much of my mental and physical energy every day. | 5 | 4 | 3 | 2 | 1 |
| My medical staff don't seem to know much about diabetes and how to manage it. | 5 | 4 | 3 | 2 | 1 |
| When I think about having to live with diabetes for the rest of my life, I feel angry, depressed, and scared. | 5 | 4 | 3 | 2 | 1 |
| My doctors do not give me enough information about how to manage my diabetes. | 5 | 4 | 3 | 2 | 1 |
| I don't test my blood glucose level as often as I should. | 5 | 4 | 3 | 2 | 1 |
| I don't seem to be managing my diabetes well (taking medication, eating well, exercising, etc.). | 5 | 4 | 3 | 2 | 1 |
| I don't feel like I'm getting enough support from my family or friends to help me manage my diabetes. | 5 | 4 | 3 | 2 | 1 |
| Diabetes seems to be taking over my life. | 5 | 4 | 3 | 2 | 1 |
| My medical staff does not take my concerns seriously. | 5 | 4 | 3 | 2 | 1 |
| I do not feel confident in my ability to manage diabetes in my daily life. | 5 | 4 | 3 | 2 | 1 |
| No matter how hard I try, I will end my life with serious diabetes complications. | 5 | 4 | 3 | 2 | 1 |
| I am not following the recommended diabetes diet sufficiently. | 5 | 4 | 3 | 2 | 1 |
| Family and friends don't understand how difficult it is to live with diabetes. | 5 | 4 | 3 | 2 | 1 |
| I feel overwhelmed by the many demands and instructions I have to follow to manage my diabetes. | 5 | 4 | 3 | 2 | 1 |
| I do not have a medical professional I can meet regularly to discuss my diabetes. | 5 | 4 | 3 | 2 | 1 |
| I lack the motivation to keep managing my diabetes well. | 5 | 4 | 3 | 2 | 1 |
| I don't get as much emotional support from family or friends as I would like. | 5 | 4 | 3 | 2 | 1 |

**Table S3. Adherence with Diabetes Medication**

| **Adherence with Diabetes Medication** | | | | | Yes | No |
| --- | --- | --- | --- | --- | --- | --- |
| Do you sometimes forget to take your diabetes medications? | | | | |  |  |
| Over the past 2 weeks, were there any days when you did not take your diabetes medicine? | | | | |  |  |
| Have you ever cut back or stopped taking your diabetes medication without telling your doctor because you felt worse when you took it? | | | | |  |  |
| When you travel or leave home, do you sometimes forget to bring along your diabetes medications? | | | | |  |  |
| Did you take your diabetes medicine yesterday? | | | | |  |  |
| When you feel like your blood glucose is under control, do you sometimes stop taking your diabetes medicine? | | | | |  |  |
| Taking medication every day is a real inconvenience for some people. Do you ever feel hassled about sticking to your diabetes treatment regimen? | | | | |  |  |
| How often do you have difficulty remembering to take all your diabetes medications? | | | | | | |
| Never | Rarely | Sometimes | Often | Always | | |

**Supplementary Figures**

**Figure S1. DoctorviceⓇ Clinic web page.**


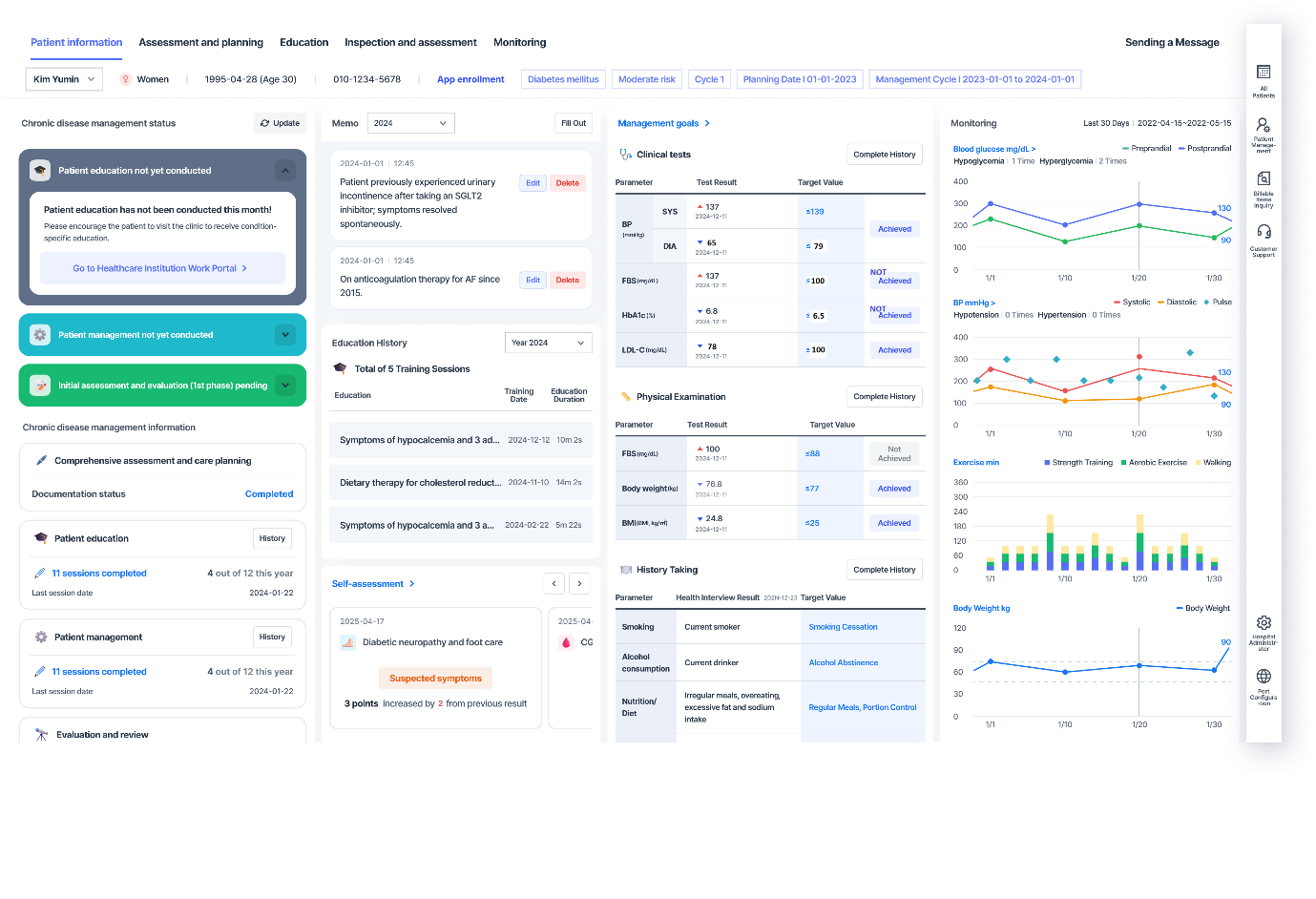


**Figure S2. Doctor’s canvas and digital contents.**


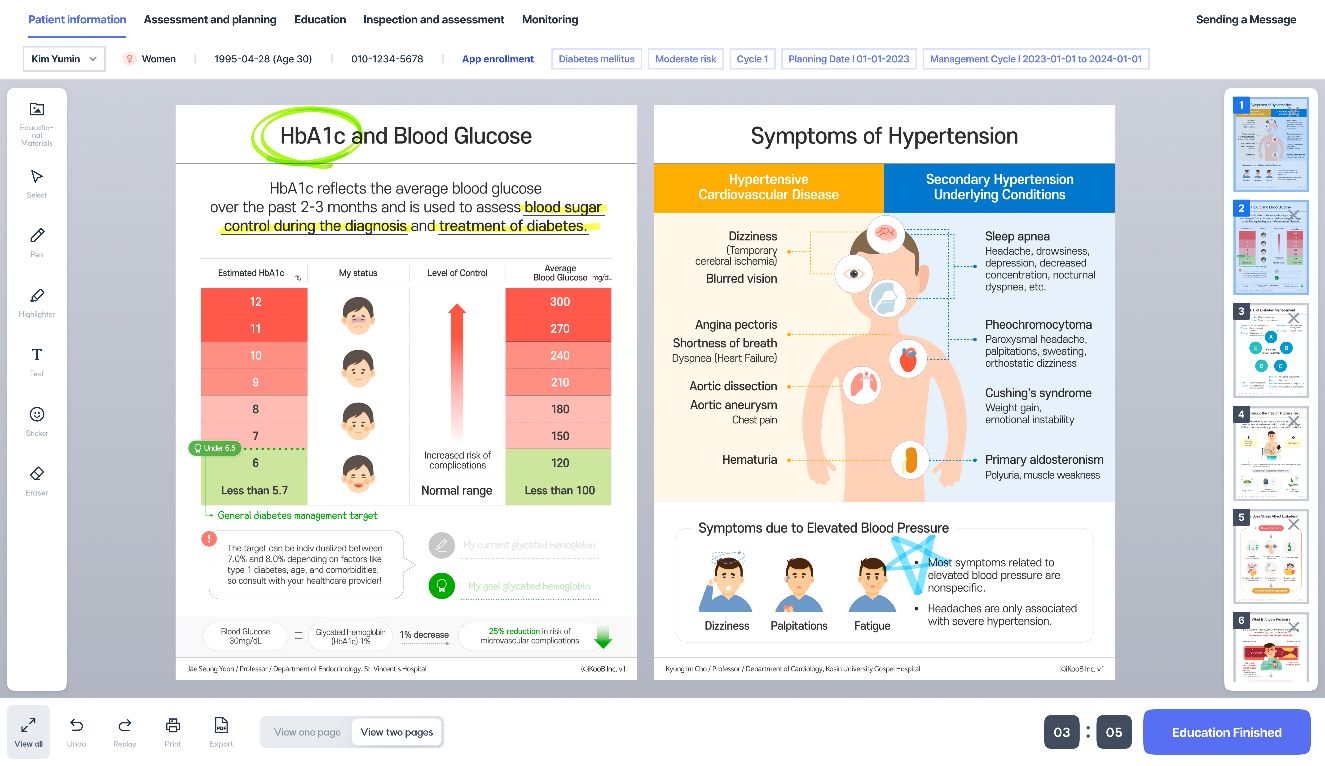


**Figure S3. Upload screen on application.**


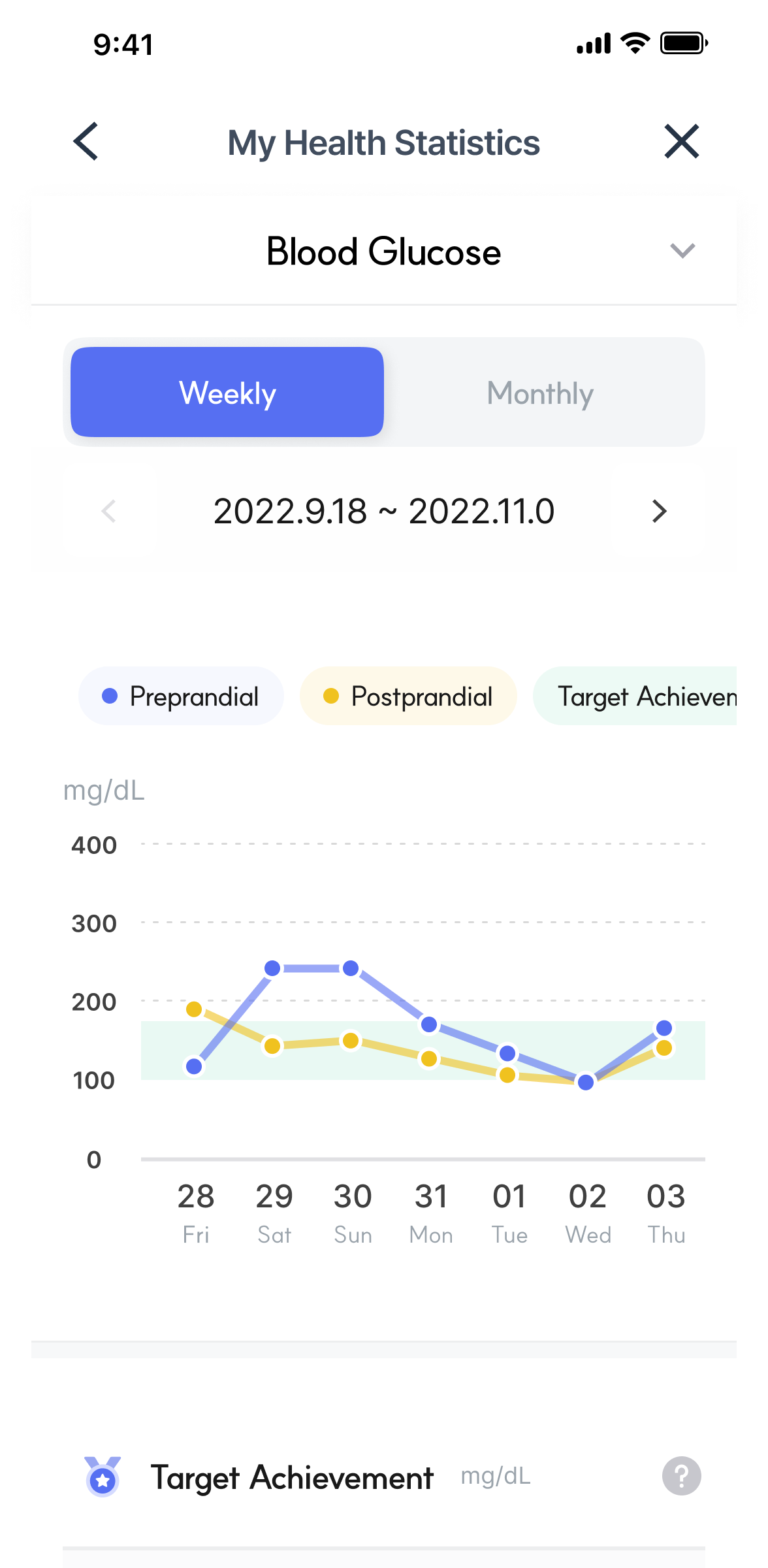

Supplement: Multimedia Appendix 1 [file mhealth_v13i1e67151_app1.docx]
